# High Strain Rate Quasi-Superplasticity Behavior in an Ultralight Mg-9.55Li-2.92Al-0.027Y-0.026Mn Alloy Fabricated by Multidirectional Forging and Asymmetrical Rolling

**DOI:** 10.3390/ma15217539

**Published:** 2022-10-27

**Authors:** Furong Cao, Huihui Shang, Nanpan Guo, Shuting Kong, Renjie Liu

**Affiliations:** 1 School of Materials Science and Engineering, Northeastern University, Shenyang 110819, China; 2 State Key Laboratory of Rolling and Automation, Northeastern University, Shenyang 110819, China; 3 AVIC Xi’an Aircraft Industry Group Company Ltd., Xi’an 710089, China

**Keywords:** magnesium-lithium alloy, multidirectional forging, asymmetrical rolling, superplasticity, microstructure, deformation mechanism

## Abstract

To explore new approaches to severe plastic deformation and the ductility of a multicomponent magnesium–lithium alloy, an ultralight microduplex Mg-9.55Li-2.92Al-0.027Y-0.026Mn alloy was made by novel multidirectional forging and asymmetrical rolling, and the superplasticity behavior was investigated by optical microscope, hot tensile test, and modeling. The average grain size is 1.9 μm in this alloy after multidirectional forging and asymmetrical rolling. Remarkable grain refinement caused by such a forming, which turns the as-cast grain size of 144.68 μm into the as-rolled grain size of 1.9 μm, is achieved. The elongation to failure of 228.05% is obtained at 523 K and 1 × 10^−2^ s^−1^, which demonstrates the high strain rate quasi-superplasticity. The maximum elongation to failure of 287.12% was achieved in this alloy at 573 K and 5 × 10^−4^ s^−1^. It was found that strain-induced grain coarsening at 523 K is much weaker than the strain-induced grain coarsening at 573 K. Thus, the ductility of 228.05% is suitable for application in high strain rate superplastic forming. The stress exponent of 3 and the average activation energy for deformation of 50.06 kJ/mol indicate that the rate-controlling deformation mechanism is dislocation-glide controlled by pipe diffusion.

## 1. Introduction

The Mg-Li alloy, the lightest nontoxic metallic alloy, has been investigated extensively in recent years [1] and has the potential for application in spaceflight, military, 3C electronic, and automobile industries on account of its excellent specific weight-to-density ratio, excellent specific strength, good damping performance, and excellent electromagnetic shielding capability. In particular, Mg-Li alloys have been used in satellites in the aerospace sector in China. When the space vehicle enters or leaves the atmosphere, it sustains high temperature [2]; when the space vehicle flies on the moon, it can sustain a severe temperature difference of 423 K [3]. Thus, it is necessary to study the high-temperature deformation behavior or superplasticity of Mg-Li alloys. In the past, studies on the superplasticity of Mg-Li binary alloy [4], Mg-Li-Zn system [5,6,7] and Mg-Li-Zn-Al system [8] alloys were reported because of the higher plasticity of the Zn element than Al element in such alloys. However, there are rare reports on the high-temperature superplasticity behavior of the Mg-Li-Al system alloy. Thus, a novel multicomponent Mg-Li-Al-Y- Mn alloy was designed, and its high-temperature behavior was studied.

Superplasticity is the capability of materials to exhibit large ductility and requires (i) fine and ultra-fine grain sizes of less than 10 μm; (ii) temperature of more than 0.5*T*_m_, where *T*_m_ is the absolute melting temperature, and a certain strain rate [9]. Superplasticity forming is especially suitable for the manufacture of complex components such as thin-wall and high-rib components. To realize superplasticity, grain refinement is essential via conventional forming and severe plastic deformation. Severe plastic deformation, as an effective grain refinement means, has attracted extensive attention of researchers over the past decades [10,11]. According to our survey, severe plastic deformation such as equal channel angular pressing [12,13,14], high-pressure torsion [15,16], friction stir processing [17,18], multidirectional forging (MDF) [19], and differential speed rolling [20] have been used in binary Mg-8Li alloy and Mg-Li-Zn alloys to achieve fine-grain and ultra-fine grain refinement and obtain superplasticity. In addition, extrusion-rolling was used in the Mg-8Li alloy to realize grain refinement and superplasticity [21]. However, to the best of our knowledge, no work is available reporting the superplasticity of a Mg-Li-Al-Y-Mn alloy processed by combining MDF and asymmetric rolling. Thus, it is necessary to utilize MDF and asymmetric rolling to achieve grain refinement and investigate the superplasticity in the present alloy.

In this work, the research contents include several aspects: (i) to fabricate a multicomponent Mg-Li-Al-Y-Mn alloy via MDF and asymmetric rolling; (ii) to investigate its microstructural phenomenon and superplasticity behavior; (iii) to establish a constitutive model and investigate the deformation mechanism at elevated temperature. It is expected that this first report on MDF and asymmetric rolling will stimulate the interests of Mg-Li alloy researchers.

## 2. Experimental Procedures

**Figure 1:**
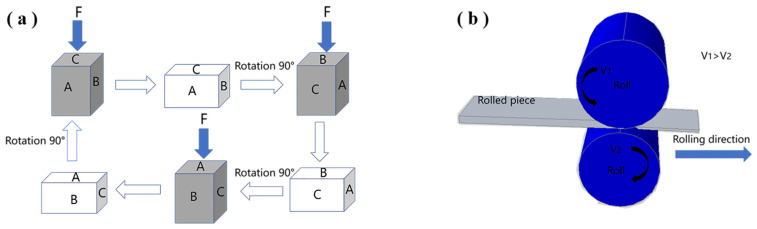
The schematic sketch of (**a**) multidirectional forging and (**b**) asymmetrical rolling. A, B, and C are pressing planes. F is the force.

The melting and casting process of alloy ingot adopts Jackson’s flux-argon atmosphere protection method. The analyzed composition of the ingot was Mg-9.55Li-2.92Al-0.027Y-0.026Mn. After milling of the ingot surface, the milled ingot was homogenized at 473 K for 16 h. The ingot was cut into billets with dimensions of 40 × 32 × 22 mm^3^. Our previous reports on multidirectional forging are available elsewhere [8,22]. The schematic sketch of multidirectional forging and asymmetrical rolling is shown in Figure 1. The cuboid billets were forged at 523 K by alternatively changing the pressing direction for six passes on a 3000 kN hydraulic press. Then, the forged billets were asymmetrically hot rolled at 523 K to plates 4 mm thick with a reduction of 81.82% and cold rolled to sheets 2 mm thick with a reduction of 50%. The asymmetrical speed ratio was 1.2. The multidirectional forging specimens for microstructural observation were taken from the central section of the cuboid. The asymmetrical rolling specimen for microstructural observation was taken from the rolled sheet along the longitudinal rolling direction.

Dog-boned specimens for tensile deformation and microstructural observation were taken along the longitudinal rolling direction and made by spark discharge processing. The specimen dimensions were 13 × 3 × 2 mm^3^. After being annealed at 448 K for 60 min and held at designated testing temperatures for 15 min, the tensile tests were performed on Shimidazu- AG-Xplus 100 kN tester in the temperature range of 423~573 K and strain rate range of 1 × 10^−2^~5 × 10^−4^ s^−1^.

Optical examination specimens were cut, ground, and polished by the conventional metallographic method. The etched solution was a solution of 10 vol.% hydrochloric acid + 90% vol.% ethyl alcohol or a solution of 5 g picric acid + 5 g acetic acid + 10 mL deionized water + 100 mL absolute alcohol. Optical microstructures were observed on Olympus-DSX500 optical microscope. Image-pro-plus (IPP) software was used to measure the grain size. The high temperature tensiled specimens were mechanically ground and polished to 80 μm. Then, discs with 3 mm diameter were punched. Chemical twin-jet and liquid nitrogen cooled ion thinning were conducted to prepare the samples for transmission electron microscopy (TEM) observation. The electrolyte was a solution of 10% HClO_4_ + 90% ethyl alcohol. The operating voltage of twin-jet was 12 V. The current was 30 mA. The temperature of electrolytic polishing was 233 K (−40 °C). The operating parameters of ion thinning were 4 kV ± 3 for 5 min, 3 kV ± 3 for 10 min, and 2 kV ± 2 for 10 min. The temperature of ion thinning ranged from 143 K (−130 °C) to 173 K (−100 °C). FEI Tecnai F30 field emission transmission electron microscope was used for dislocation observation.

## 3. Results

### 3.1. Initial Thermomechanical Processing Microstructures

**Figure 2:**
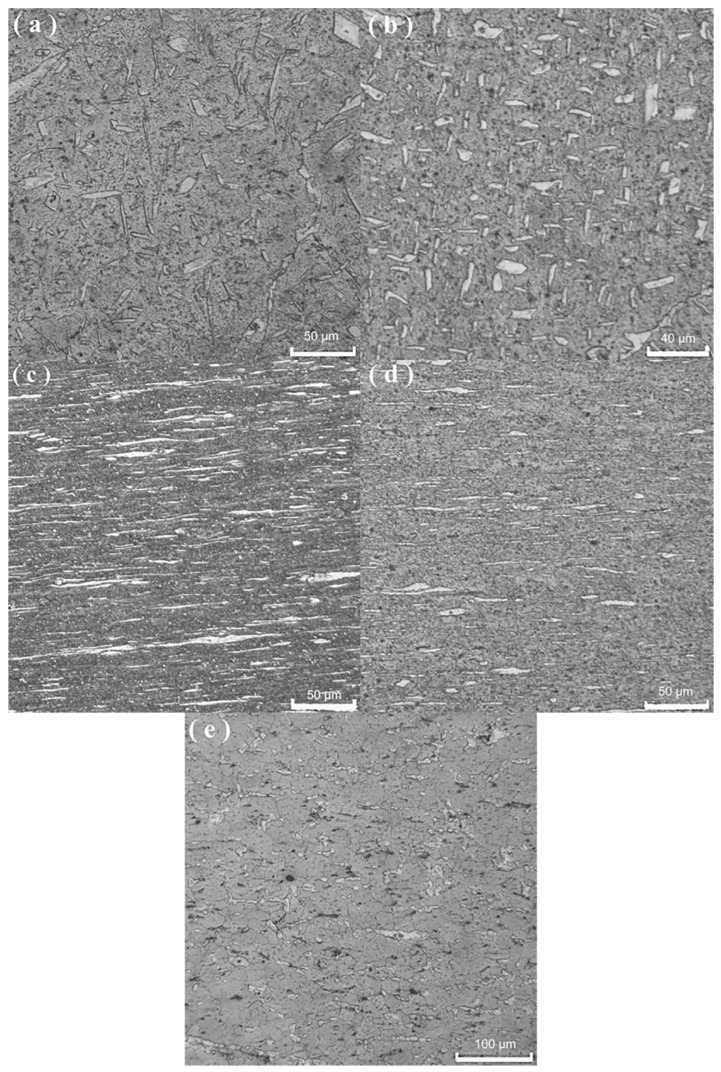
Optical microstructures of the present alloy under different processing states: (**a**) as-cast state, (**b**) 6-pass multidirectional forging (523 K), (**c**) multidirectional forging (523 K) asymmetric hot rolling (523 K) cold rolling state, (**d**) 448 K × 60 min annealing, and (**e**) gripping section, 573 K × 15 min holding.

Figure 2 shows the optical microstructures of the present alloy under different processing states. As shown in Figure 2a, the as-cast structure consists of white acicular and plate-like α-Mg phase and gray β-Li phase, where the α-Mg phase is distributed in the matrix of the β-Li phase. The average grain size is 144.68 μm. The measurement method of average grain size is to use IPP software and obtain the linear intercept grain size. As shown in Figure 2b, after six-pass multidirectional forging, both phases are refined due to shear stress caused by the pressing stress that keeps changing its loading direction. The average grain size is 11.72 μm. As shown in Figure 2c, after multidirectional forging (523 K), asymmetric hot rolling (523 K), cold rolling, grains were greatly refined under imposed rolling stress, and banded or elongated grains are clearly visible. The average grain size measured by IPP software is 1.9 μm along the vertical rolling direction. As shown in Figure 2d, the banded α-Mg grains shorten, and some equiaxed grains appear due to static recrystallization in the alloy sheet annealed at 448 K for 60 min. The average grain size is 4.14 μm. As shown in Figure 2e, after the sample is kept at 573 K for 15 min, pronounced grain coarsening occurs because of high-temperature grain boundary migration. The average grain size in the gripping section of the specimen is 18.26 µm.

### 3.2. High-Temperature Tensile Mechanical Properties and Microstructures

#### 3.2.1. High-Temperature Tensile Mechanical Properties

**Figure 3:**
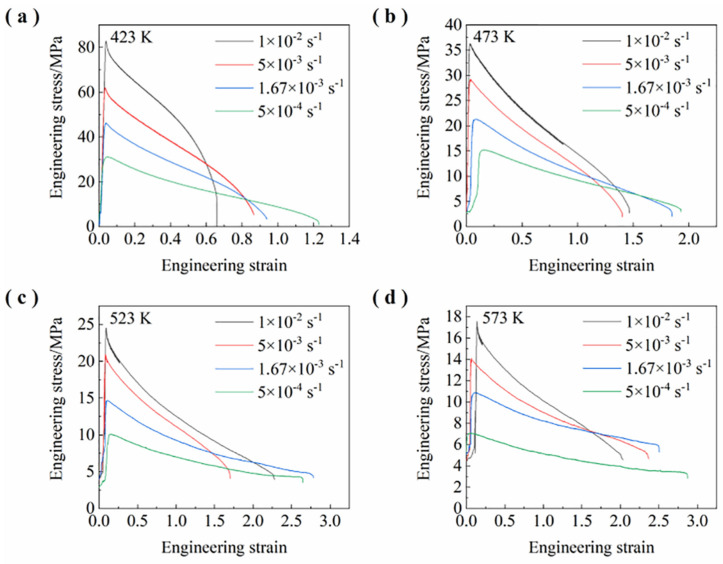
Engineering stress–strain curves of this alloy at different temperatures and strain rates: (**a**) 423 K, (**b**) 473 K, (**c**) 523 K, and (**d**) 573 K.

Figure 3 shows the engineering stress–strain curves of this alloy at different temperatures and strain rates. In most cases, the engineering strain or elongation to failure increases with decreasing strain rates from 1 × 10^−2^ to 5 × 10^−4^ s^−1^. The engineering stress decreases with the increase in tensile temperatures from 423 to 573 K. This is because, with the decrease in strain rate and the increase in temperature, the tensile time prolongs and thermal activation accelerates, dislocation density decreases, and stress decreases. As shown in Figure 3b, the ductility or elongation of 146.39% is obtained at 473 K and 1 × 10^−2^ s^−1^, which demonstrates the low temperature and high strain rate quasi-superplasticity. As shown in Figure 3c, the ductility or elongation of 228.05% is obtained at 523 K and 1 × 10^−2^ s^−1^, which demonstrates the high strain rate quasi-superplasticity. As shown in Figure 3d, the maximum ductility or elongation of 287.12% was obtained in this alloy at 573 K and 5 × 10^−4^ s^−1^.

#### 3.2.2. High-Temperature Tensile Microstructures

**Figure 4:**
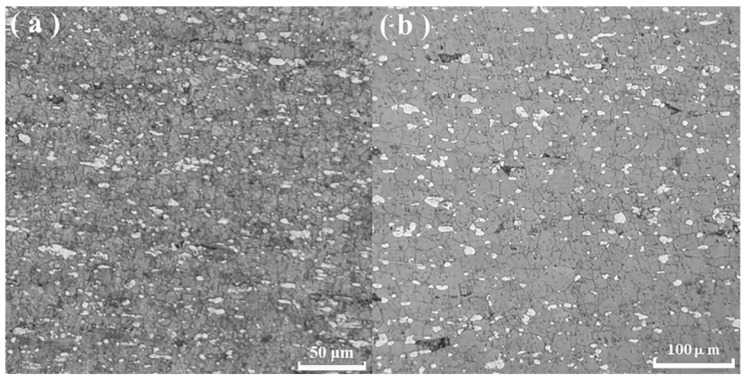
Microstructures of the gauge section in this alloy at (**a**) 523 K and 1 × 10^−2^ s^−1^ and (**b**) 573 K and 5 × 10^−4^ s^−1^.

Figure 4 presents the microstructures of the gauge section in this alloy at 523 K and 1 × 10^−2^ s^−1^ and 573 K and 5 × 10^−4^ s^−1^. Average grain sizes under both conditions are 6.42 and 22.32 µm, respectively. Compared to the grain size of 4.14 µm in Figure 2c, slight dynamic grain coarsening occurs at 523 K and 1 × 10^−2^ s^−1^. In addition, compared to Figure 2d, pronounced dynamic grain coarsening occurs at 573 K and 5 × 10^−4^ s^−1^. This means that 287.12% elongation is obtained in this coarse grained microstructure. Furthermore, the average grain sizes of α-Mg and β-Li phases are 2.03 and 6.91 μm, respectively, at 523 K and 1 × 10^−2^ s^−1^. The average grain sizes of α-Mg and β-Li phases are 9.64 and 26.27 μm, respectively, at 573 K and 5 × 10^−4^ s^−1^. As the temperature increases and the strain rate decreases, the average grain sizes of the dual phase increase. This indicates the occurrence of phase coarsening.

**Figure 5:**
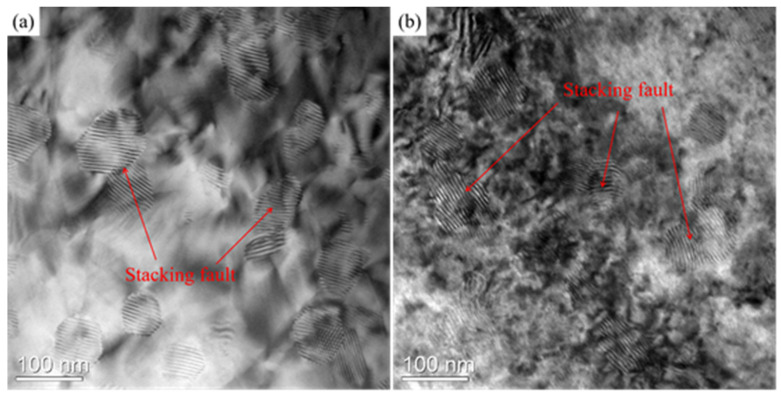
TEM images of stacking faults in the present alloy at (**a**) 523 K and 1 × 10^−2^ s^−1^ and (b) 573 K and 5 × 10^−4^ s^−1^.

Figure 5 presents the TEM images of stacking faults in the present alloy at 523 K and 1 × 10^−2^ s^−1^ and 573 K and 5 × 10^−4^ s^−1^. Some stacking faults exist in the alloy. Since the samples for TEM examination have been exposed to the ambient environment for 365 days, with dislocations existing in high energy and non-equilibrium state, when the high temperature tensile test was conducted, they reacted and dissociated to become the current low-energy and equilibrium states as shown in Figure 5. That may be the cause that the stacking faults appear. This indicates the activity of dislocation glide when the high temperature tensile test was performed. This interesting discovery of stacking faults in the present alloy is the first time in the study of a Mg-Li alloy system deformed at elevated temperature.

#### 3.2.3. Strain Rate Sensitivity Index (*m* Value)

**Figure 6:**
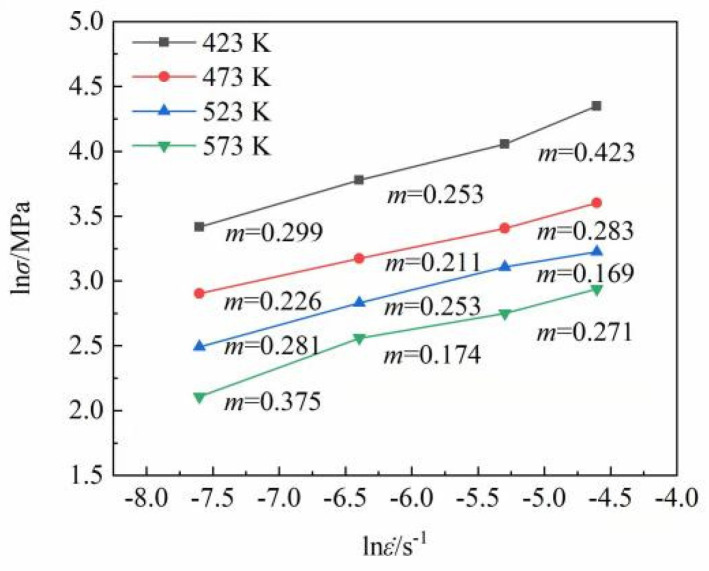
The strain rate sensitivity index *(m* value) under different conditions: Log true stress–log strain rate curves.

Figure 6 presents the variation of the strain rate sensitivity index (*m* value) under different conditions. *m* = $\partial \ln\sigma /\partial \ln\dot{\varepsilon }$, where *σ* is the true stress and $\dot{\varepsilon }$ is the strain rate. The *m* values range from 0.169 to 0.423, most of which lie between 0.2 and 0.3, indicating that the dominant deformation mechanism is dislocation creep. The *m* value of 0.375 (stress exponent *n* = 1/*m* = 2.66 ≈ 3) corresponds to the maximum elongation to failure of 287.12%, indicative of the occurrence of quasi-superplasticity or superplasticity-like behavior. The stress exponent *n* = 2.66 ≈ 3 reveals that dislocation viscous glide governs the rate-controlling process under this condition.

### 3.3. Establishment of Power-Law Constitutive Equation at Elevated Temperature

The power-law constitutive equation at elevated temperature is generally expressed as [23]

(1) $$\dot{\varepsilon }=\frac{AD_{0}Gb}{kT}{\left(\frac{b}{d}\right)}^{p}{\left(\frac{\sigma -\sigma_{0}}{G}\right)}^{n}\exp\left(-\frac{Q}{RT}\right)$$

where $\dot{\varepsilon }$ is the steady-state deformation rate, A is a dimensionless constant, *G* is the shear modulus, a function of temperature, *b* is the magnitude of Burgers vector of dislocation, *k* is Boltzmann’s constant, *T* is the absolute temperature, *d* is the grain size, *p* is the grain size exponent, *σ* is the applied stress, $\sigma_{0}$ is the threshold stress, *n* is the stress exponent (1/*m*, *m*-strain rate sensitivity index), *D*_0_ is the frequency factor for diffusion, *Q* is the activation energy for deformation, and *R* is the universal gas constant. Here, power-law constitutive modeling is performed to elucidate the deformation mechanism at elevated temperature and is suitable for the application in superplastic forming process control. In order to determine the threshold stress, *n*-value, and *Q*-value, true stress and true strain formulae are used on the basis of Figure 3. True stress = Engineering stress × (1 + Engineering strain); True strain = Ln (1 + Engineering strain).

**Figure 7:**
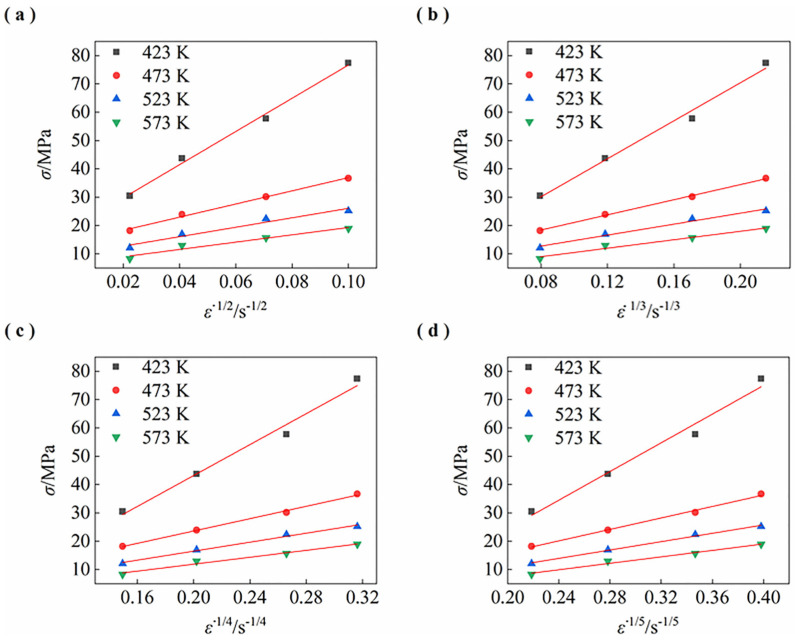
The linear fitting of σ—$\dot{\varepsilon }$ ^1/*n*^ relation in this alloy: (**a**) *n* = 2; (**b**) *n* = 3; (**c**) *n* = 4; (**d**) *n* = 5.

Figure 7 shows the linear fitting of σ—$\dot{\varepsilon }$^1/*n*^ relation in this alloy to determine the threshold stress and stress exponent. At the true strain of 0.2, the values of threshold stress $\sigma_{0}$ are determined using linear fitting of σ—$\dot{\varepsilon }$^1/*n*^ relation. When *n* = 4 and 5, the threshold stresses become negative. Hence, *n* = 4 and *n* = 5 are excluded. When *n* is 3, the determination coefficient, *R*^2^, is 0.9858 with the best correlation, which is higher than the determination coefficient, *R*^2^, of 0.9759 when *n* = 2. Thus, the true stress exponent is determined to be 3.

As per Equation (1), the deformation activation energy is given by

(2) $$Q=R{\frac{\partial \left[\ln\left(\sigma^{n}G^{1-n}T^{-1}d^{-p}\right)\right]}{\partial \left(T^{-1}\right)}|}_{\;\dot{\varepsilon }}$$

As dislocation creep is predominant in this alloy, *p* is equal to zero [24].

Young’s modulus of Mg is given by *E* = 48,700 − 8.59*T* − 0.0195*T*^2^ [25]. The relationship between Young’s modulus *E*, Poisson’s ratio *υ*, and shear modulus *G* is given by

(3) $$G=\frac{48,700-8.59T-0.0195T^{2}}{2\left(1+\upsilon \right)}$$

where Poisson’s ratio *υ* of Mg is 0.28 [26].

**Figure 8:**
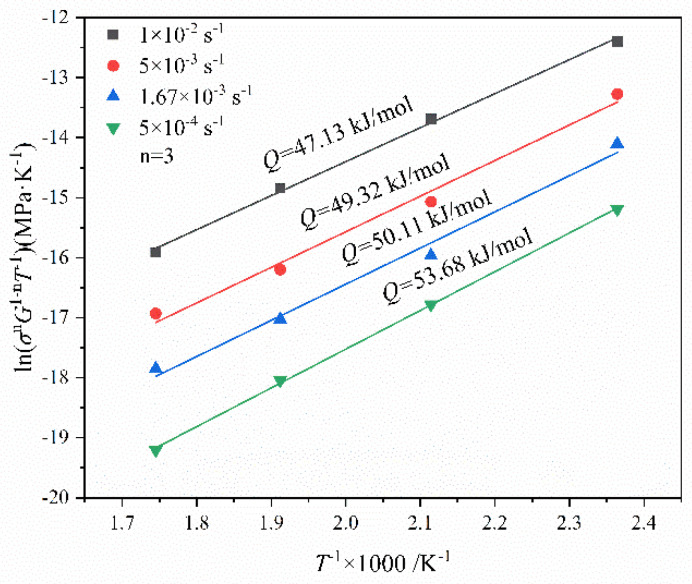
Fitting lines of $\ln\left(\sigma^{n}G^{1-n}T^{-1}\right)$—1000/*T* at various strain rates.

Figure 8 shows the fitting curves of $\ln\left(\sigma^{n}G^{1-n}T^{-1}\right)-1/T$ at various strain rates. The activation energy for high temperature deformation of the present alloy is in the range of 47.13~53.68 kJ/mol. Average experimental activation energy is 50.56 kJ/mol.

**Figure 9:**
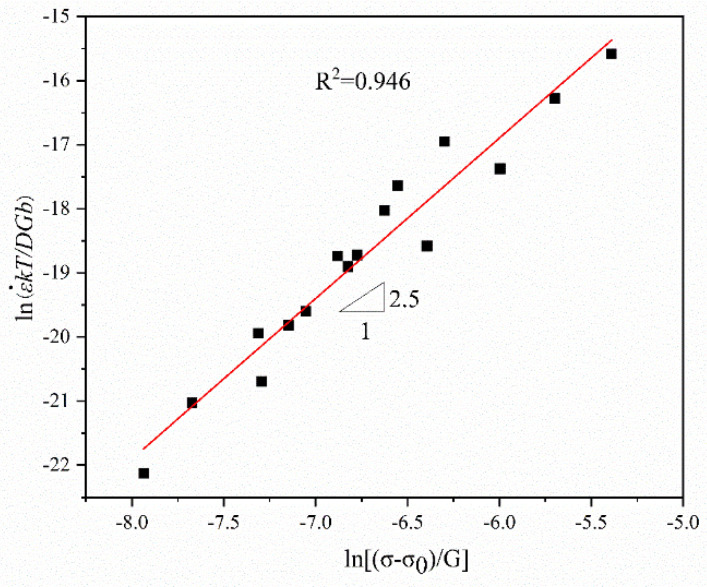
Normalized curve of $\ln\left(\dot{\varepsilon }kT/DGb\right)-\ln\left[\left(\sigma -\sigma_{0}\right)/G\right]$. The black squares are data points.

Figure 9 shows the normalized curve of $\ln\left(\dot{\varepsilon }kT/DGb\right)-\ln\left[\left(\sigma -\sigma_{0}\right)/G\right]$. The slope of the linear fitting line is 2.50327 (≈3). The intercept of the fitting line is lnA (=−1.87561). Hence, A  = 0.153261. The determination coefficient, *R*^2^, is 0.946. Thus, the power-law constitutive equation is obtained as the following:

(4) $$\dot{\varepsilon }=1.532\times 10^{-5}\frac{Gb}{KT}{\left(\frac{\sigma -\sigma_{0}}{G}\right)}^{2.5}\exp\left(\frac{-50,561.13}{RT}\right)$$

## 4. Discussion

### 4.1. Analysis of the Processing Principle of Our MDF + Asymmetrical Rolling Approach

The method of MDF + asymmetrical rolling is proposed in this manuscript and put into effect via experimental forming. The total imposed strain during the MDF + asymmetrical rolling processing is 6.2. The principle behind the combined forming is that the accumulated strain, 6.2, through MDF + asymmetrical rolling is much larger than the accumulative strain of MDF, 3.6, and the strain of asymmetrical rolling, 2.6. Here, the strain of asymmetrical rolling, 2.6, is calculated using the true strain formula in reference [27]. Thus, the grain refinement of MDF + asymmetrical rolling is superior to the grain refinement of simple MDF and simple asymmetrical rolling. That is the advantage of MDF + asymmetrical rolling.

Compared to the average grain size of 5.5 μm in our previous work [28] in Mg-6.4Li-3.6Zn-0.37Al-0.36Y alloy and the average grain size of 3.75 μm in our previous work [8] in Mg-10.2Li-2.23Zn-2.1Al-0.2Sr processed by MDF+ symmetrical flat rolling, the average grain size in Figure 2b is 1.9 μm in the present alloy (Mg-9.55Li-2.92Al-0.027Y-0.026Mn) fabricated by MDF + asymmetrical rolling. As shown in Figure 2b, grains are fragmented and refined after MDF + asymmetrical rolling. This reveals that remarkable grain refinement is achieved due to novel MDF+ asymmetrical rolling. This is because compared to symmetrical flat rolling, asymmetrical rolling exerts more shear action on the rolled piece during the rolling deformation process. The intense shear imposed by the asymmetrical rolling intensifies the grain fragmentation and refinement. Here, it is noted that the average grain size is measured by IPP software and is a statistical result of grain band width because the rolling grain size is usually expressed by the average grain band width. In addition, asymmetrical rolling results in fine equiaxed grains in AZ (Mg-Al-Zn) magnesium alloys, but leads to banded or elongated grains instead of equiaxed grains in the Mg-Li alloy such as the present (Mg-9.55Li-2.92Al-0.027Y-0.026Mn) alloy.

### 4.2. Analysis of Dynamic Grain Coarsening after Tension

In recent years, the issue of grain coarsening including static grain coarsening [29,30,31] and dynamic grain coarsening [32,33,34] has attracted the attention of researchers in superplastic aluminum alloys and zinc-0.8Ag alloys, but according to our survey, only a few reports are available regarding dynamic grain coarsening in superplastic magnesium alloys. In particular, little information is available in superplastic Mg-Li based alloys except our for previous work [35]. Here, dynamic grain coarsening means deformation-induced grain coarsening or strain-induced grain coarsening. With the increase in strain, grain boundary and phase boundary migration increase, and grain becomes coarse, which is called strain-induced grain coarsening.

As shown in Figure 4, compared to the initial microstructures in Figure 2c,d, strain-induced grain coarsening appears in the tensile deformation alloy. Due to short-time weak coarsening (grain size 6.42 µm) at a high strain rate of 1 × 10^−2^ s^−1^ and 523 K, the elongation to failure of 228.05% is obtained at 523 K and 1 × 10^−2^ s^−1^. It is found that strain-induced grain coarsening at 523 K is much weaker than the strain-induced grain coarsening at 573 K. Thus, the ductility of 228.05% is especially suitable for application in high strain rate quasi-superplastic forming. Furthermore, due to pronounced grain coarsening (grain size 22.32 µm) at 573 K, the elongation of 287.12% is obtained in this alloy at 573 K and 5 × 10^−4^ s^−1^. The causes of grain coarsening are analyzed as follows.

Firstly, the experimental phase proportion of α-Mg phase to β-Li phase, which is 15.59:84.41, indicates that the present alloy is a β-Li phase-dominated alloy with a small volume fraction of α-Mg phase. As such, the capability of hard α-Mg phase in restricting the coarsening of soft β-Li phase is weaker at 573 than at 523 K. Secondly, based on the report on the diffusivities of α-Mg and β-Li phases [36], the diffusivity or mobility of the β-Li phase is much faster than the diffusivity or mobility of the α-Mg phase. Hence, the β-Li-dominated alloy is prone to grain coarsening at higher temperature. Thirdly, according to Jin et al.’s report [37], the product of grain boundary width multiplied by grain boundary diffusivity for Al abides by the following relation:

(5) $$\delta D_{\mathrm{gb}}=5\times 10^{-4}\exp\left(-\frac{84,000}{RT}\right)\;$$

where δ is the grain boundary width, (=2*b*), *b* is the magnitude of Burgers vector of dislocation, 2.86 × 10^−10^ m (Al), *D*_gb_ is the grain boundary diffusivity of Al, *R* is the gas constant, and *T* is the absolute temperature. Thus, the grain boundary diffusivity of Al at 573 K is calculated to be 1.92 × 10^−12^ m^2^ s^−1^. The grain boundary diffusivity of Mg at 573 K is 2.88 × 10^−11^ m^2^ s^−1^ [36]. As the grain boundary mobility *M*_gb_ is equal to *D*_gb_/*kT* based on the Einstein equation, where *k* is Boltzmann’s constant, *M*_gb_ (Mg)/*M*_gb_ (Al) = *D*_gb_ (Mg)/*D*_gb_ (Al). Hence, at 573 K, *M*_gb_ (Mg)/*M*_gb_ (Al) = *D*_gb_ (Mg)/*D*_gb_ (Al) = 15:1. Our quantitative calculation of Al and Mg diffusivity reveals that the grain boundary diffusivity or mobility of Mg is 15 times as much as that of Al. Higher mobility leads to higher boundary migration. Higher boundary migration results in strain-induced grain coarsening. This means that strain-induced grain coarsening in Mg alloys is higher than that in Al alloys. It is not surprising that strain-induced grain coarsening is a common phenomenon and feature in Mg alloys at a certain temperature, regardless of the strain-induced grain coarsening in the present Mg-9.55Li-2.92Al-0.027Y-0.026Mn alloy. The aforementioned viewpoint is supported by our result in this Mg-Li-Al-Y-Mn alloy, Kim et al.’s thought [38], and Figueiredo-Langdon’s grain coarsening evidence [39] in ZK60 (Mg-Zn-Zr) magnesium alloy tensiled at 493 K. Fourthly, as the contents of Y and Mn elements are 0.027 and 0.026 wt %, respectively, with low concentration in this alloy, Zener pinning cannot be effectively realized through the intermetallic compounds formed by Al and both elements, the grain boundary (α-Mg/α-Mg, β-Li/β-Li) migration and (α-Mg/β-Li) phase boundary migration occur, and strain-induced grain coarsening occurs.

In addition, the superplasticity of Mg-Li-Al system alloy and binary Mg-Li alloy are analyzed. Dutkiewicz et al. [40] have recently reported the superplasticity of the Mg-9Li-2Al-0.5Sc alloy fabricated by extrusion and cyclic forging. They have obtained the superplastic elongations between 150 and 190% at 423 K and claimed that the superplasticity of the Mg-Li-Al system alloy is lower than the superplasticity of the binary Mg-Li alloy. This is consistent with our experimental results that the elongation of the Mg-9.55Li-2.92Al-0.027Y-0.026Mn alloy is lower than that of the Mg-8Li alloy [36]. This is because the addition of Al, Y, and Mn elements to binary Mg-9.55Li alloy increases the deformation resistance of intragranular slip in the matrix, raises the flow stress, and disfavors grain boundary sliding. Meanwhile, the phase proportion in this alloy is not adjacent to 50:50 and does not induce a superplastic crane effect. However, for the Mg-8Li alloy without these elements, the crane effect, a phenomenon that the maximum superplastic elongation or grain boundary sliding is achieved in dual phase alloy under the 50:50 phase proportion, is easily realized in this dual-phase alloy with 50:50 phase proportion.

### 4.3. Deformation Mechanism at Elevated Temperatures

The activation energy for deformation and stress exponents were determined so as to judge the deformation mechanism at elevated temperature. The phase proportion of the α-Mg phase to β-Li phase is calculated to be 16.30:83.70 as per the binary Mg-Li phase diagram [41], and the experimental phase proportion of α-Mg phase to β-Li phase is measured to be 15.59:84.41, indicating that this alloy is a β-Li phase-dominated multicomponent alloy, which is consistent with the microstructures in Figure 2. To obtain the theoretical activation energy according to our previous model [36], the following relations are presented in the Mg-9.55Li-2.92Al-0.027Y-0.026Mn alloy:

(6) $$D_{\mathrm{gb}}=0.84\times 10^{-4}\exp\left(-\frac{8256.05}{T}\right)+0.16\times 10^{-4}\exp\left(-\frac{8106.45}{T}\right)\;$$

(7) $$D_{l}=0.84\times 10^{-4}\exp\left(-\frac{1.55\times 10^{4}}{T}\right)+0.16\times 2.5\times 10^{-4}\exp\left(-\frac{1.24\times 10^{4}}{T}\right)$$

where *D*_gb_ is the grain boundary diffusivity, *D*_l_ is the lattice diffusivity, and *T* is the absolute temperature in Kelvin. Due to *D*_p_ = *D*_gb_ [42], where *D*_p_ is the coefficient of pipe diffusion or pipe diffusivity, in consideration of *D* = *D*_0_ exp(−*Q*/*RT*), where *D*_0_ = 1 × 10^−4^ m^2^ s^−1^ [43], and Equations (6) and (7), Table 1 is obtained. In terms of the above-mentioned grain boundary diffusivity of Mg, 2.88 × 10^−11^ m^2^ s^−1^ and the pipe diffusivity of 5.79 × 10^−11^ m^2^ s^−1^ at 573 K in Table 1, the grain boundary (or pipe) diffusivity or mobility of the present alloy is two times as much as that of Mg. As we know, the grain-coarsening velocity or boundary-migration velocity is directly proportional to the mobility. This means that the grain-coarsening velocity of the present alloy is two times as much as that of Mg at 573 K. Because of grain coarsening at 573 K, grain boundary sliding is hindered, and intragranular sliding is enhanced. As a result, ductility decreases. This indirectly indicates that the appropriate quasi-superplastic deformation temperatures for the present Mg-9.55Li-2.92Al-0.027Y-0.026Mn alloy is from 473 to 523 K in which strain-induced grain coarsening is not obvious.

It is noted in Table 1 that the theoretical pipe diffusion activation energy, *Q*_p_, is 68.4 kJ/mol, and the theoretical lattice diffusion activation energy, *Q*_l_, is 107 kJ/mol in the temperature range of 473–573 K. As shown in Section 3.3, the average experimental activation energy is 50.56 kJ/mol, which is close to the theoretical activation energy of pipe diffusion, 68.4 kJ/mol. This reveals that pipe diffusion governs the diffusion process. Meanwhile, as shown in Section 3, the stress exponent is determined to be 3, indicating that dislocation viscous glide governs the rate-controlling process. In terms of available reports on dislocation viscous glide or solute drag creep in solid solution-based aluminum alloys [44,45,46,47] and quasi-single phase magnesium alloys [48] deformed at elevated temperature, the occurrence of dislocation viscous glide or solute drag creep results from the interaction of solutes and dislocations. However, the appearance of dislocation glide in the β-phase-dominated Mg-9.55Li-2.92Al-0.027Y-0.026Mn alloy is a new discovery. Thus, the deformation mechanism at elevated temperature is dislocation glide controlled by pipe diffusion.

**Figure 10:**
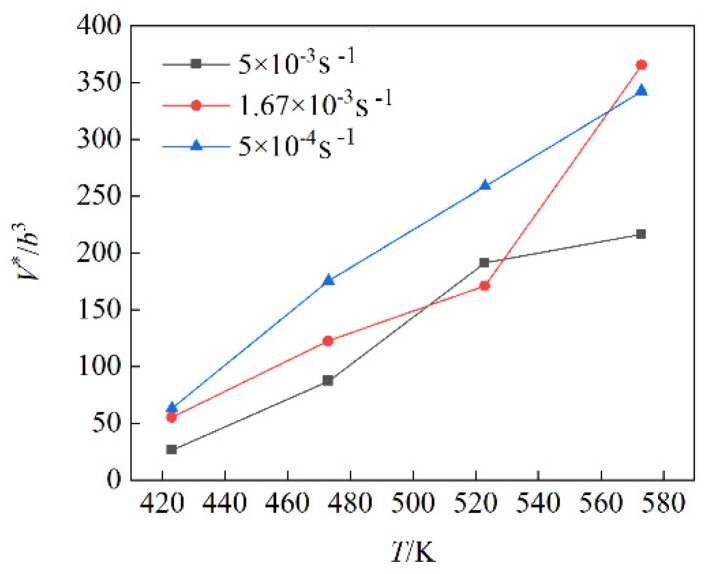
Normalized activation volume as a function of temperature at different strain rates.

Furthermore, the activation volume is estimated to judge the deformation mechanism at elevated temperature. The activation volume,$\;V^{*}$, is given by the following formula [49]

(8) $$V^{*}\equiv M_{T}kT{\left(\partial \ln\dot{\varepsilon }/\partial \sigma \right)}_{T}=M_{T}kT{\left[\ln\left({\dot{\varepsilon }}_{2}/{\dot{\varepsilon }}_{1}\right)/\Delta \sigma \right]}_{T}$$

where *M_T_* is Taylor factor, =4.5 [50] for the equiaxed grain microstructure in Figure 4, *k* is Boltzmann’s constant, *T* is the absolute temperature, $\dot{\varepsilon }$ is the strain rate, *σ* is the true stress, and ${\left[\ln\left({\dot{\varepsilon }}_{2}/{\dot{\varepsilon }}_{1}\right)/\Delta \sigma \right]}_{T}$ indicates the variation in logarithmic strain rate divided by the yield stress difference at constant temperature. *b*^3^, where *b* is the magnitude of Burgers vector of dislocation, is considered as a volume unit. Here, the *b* value of Mg is 3.21 × 10^−10^ m. The V*/*b*^3^ is taken as the normalized activation volume. Figure 10 shows the normalized activation volume as a function of temperature at different strain rates. There are three curves in Figure 10 because the calculation of activation volume in Equation (8) involves the varying strain rate or jump strain rate. The normalized activation volume increases with the increase in deformation temperature. It is reported [51] that when the grain size ranges from 6 to 40 μm, V* = 100 − 300*b*^3^, in this case, trans-granular dislocation slip occurs, but when V* < 1 − 10*b*^3^, grain boundary sliding occurs in nanometer material. The experimental grain sizes of 6.42 and 22.32 μm fall into the range of 6–40 μm. Since the ratio of V*/*b*^3^ is in the range of 25–366 *b*^3^, as shown in Figure 10 at different temperatures, dislocation slip governs the deformation mechanism. According to what has been described above, we can conclude that the deformation mechanism at elevated temperature is dislocation glide or slip.

To further validate the dislocation activity, an estimation was made to calculate the number of dislocations inside a grain at 523 K and 1 × 10^−2^ s^−1^. The number of dislocations inside a grain is given by the following relation [52]:

(9) N=1.81[1−ν][dσ/(Gb)]

where *N* is the number of dislocations, ν is Poisson’s ratio, 0.28 for Mg, *d* is the grain size, *d* = 6.42 μm (Figure 4a), σ is the true stress, 16.4 MPa is determined by the true stress (strain)–engineering stress (strain) relation in Section 3.2.1, *G* is the shear modulus, 15,189 MPa (Equation (3)), and *b* is the magnitude of Burgers vector, 3.21 × 10^−10^ m for Mg. Thus, *N* = 28.14 ≈ 29. There are 29 pieces of dislocations inside a grain under this condition. In consideration of experimental evidence of stacking faults dissociated from the dislocation reaction in Figure 4a, theoretical estimation and experimental evidence support the occurrence of dislocation glide. Moreover, Equation (9) was validated in our previous report on the hot-compressed Al-Mg-Er-Zr alloy [53] and is convincing. In terms of aforementioned facts and analyses, the deformation mechanism at elevated temperature is found to be dislocation glide controlled by pipe diffusion.

## 5. Conclusions

(1) An ultralight microduplex Mg-9.55Li-2.92Al-0.027Y-0.026Mn alloy was made by novel multidirectional forging and asymmetrical rolling. The average grain size was 1.9 μm in the present alloy fabricated by multidirectional forging + asymmetrical rolling. Remarkable grain refinement caused by such a forming, which turns the as-cast grain size of 144.68 μm into the as-rolled grain size of 1.9 μm, was achieved.

(2) The elongation to failure of 228.05% was obtained at 523 K and 1 × 10^−2^ s^−1^, which demonstrates the high strain rate quasi-superplasticity. The maximum elongation to failure of 287.12% was demonstrated in this alloy at 573 K and 5 × 10^−4^ s^−1^. It was found that strain-induced grain coarsening at 523 K is much weaker than the strain-induced grain coarsening at 573 K. Thus, the ductility of 228.05% is suitable for application in high strain rate superplastic forming. Theoretical analysis of atomic diffusion shows that the grain-coarsening velocity of the present alloy was two times as much as that of Mg at 573 K. This indicates that the appropriate quasi-superplastic deformation temperatures for the present Mg-9.55Li-2.92Al-0.027Y-0.026Mn alloy is from 473 to 523 K in which strain-induced grain coarsening is not obvious.

(3) The power-law constitutive equation was established. The stress exponent was determined to be 3. The average activation energy for deformation was 50.06 kJ/mol, which is close to the theoretical activation energy of pipe diffusion, 68.4 kJ/mol. The results of estimation of stress exponent, activation energy, and activation volume indicate that the rate-controlling deformation mechanism is dislocation glide controlled by pipe diffusion.

## Figures and Tables

**Table 1:** The values of diffusivity *D* and activation energy *Q* at different temperatures in the present alloy.

*T* (K)	*D*_p_ (m^2^/s)	*Q*_p_ (kJ/mol)	*D*_l_ (m^2^/s)	*Q*_l_ (kJ/mol)
423	3.57 × 10^−13^	68.405	7.5 × 10^−18^	106.283
473	2.79 × 10^−12^	68.405	1.7 × 10^−16^	106.573
523	1.47 × 10^−11^	68.410	2.0 × 10^−15^	107.120
573	5.79 × 10^−11^	68.419	1.6 × 10^−14^	107.454

## Data Availability

The data presented in this study are available on request from the corresponding author.
